# Gremlin inhibits UV-induced skin cell damages via activating VEGFR2-Nrf2 signaling

**DOI:** 10.18632/oncotarget.12454

**Published:** 2016-10-04

**Authors:** Chao Ji, Jin-wen Huang, Qiu-yun Xu, Jing Zhang, Meng-ting Lin, Ying Tu, Li He, Zhi-gang Bi, Bo Cheng

**Affiliations:** ^1^ Department of Dermatology, The First Affiliated Hospital of Fujian Medical University, Fuzhou 350005, Fujian, China; ^2^ Department of Dermatology, The First Affiliated Hospital of Kunming Medical University, Yunnan Provincial Institute of Dermatology, Kunming 650032, Yunnan, China; ^3^ Department of Dermatology, BenQ Medical Center, Nanjing Medical University, Nanjing 210019, Jiangsu, China

**Keywords:** ultra violet (UV), skin cell damage, gremlin, VEGFR2, Nrf2

## Abstract

Ultra Violet (UV) radiation induces reactive oxygen species (ROS) production, DNA oxidation and single strand breaks (SSBs), which will eventually lead to skin cell damages or even skin cancer. Here, we tested the potential activity of gremlin, a novel vascular endothelial growth factor (VEGF) receptor 2 (VEGFR2) agonist, against UV-induced skin cell damages. We show that gremlin activated VEGFR2 and significantly inhibited UV-induced death and apoptosis of skin keratinocytes and fibroblasts. Pharmacological inhibition or shRNA-mediated knockdown of VEGFR2 almost abolished gremlin-mediated cytoprotection against UV in the skin cells. Further studies showed that gremlin activated VEGFR2 downstream NF-E2-related factor 2 (Nrf2) signaling, which appeared required for subsequent skin cell protection. Nrf2 shRNA knockdown or S40T dominant negative mutation largely inhibited gremlin-mediated skin cell protection against UV. At last, we show that gremlin dramatically inhibited UV-induced ROS production and DNA SSB formation in skin keratinocytes and fibroblasts. We conclude that gremlin protects skin cells from UV damages via activating VEGFR2-Nrf2 signaling. Gremlin could be further tested as a novel anti-UV skin protectant.

## INTRODUCTION

A large proportion of the newly-diagnosed cancers are skin cancers [1–4]. Solar Ultra Violet (UV) radiation is the well-established pathological cause of skin cancer [5]. It is known that UV radiation in skin cells, mainly keratinocytes and fibroblasts, will induce oxidative stresses and DNA damages, activation of several signal transduction pathways, and transcriptional expression of genes involved in tumor initiation and progression [6, 7]. It is therefore vital to develop novel anti-UV agents for skin cancer prevention.

Gremlin is a highly-conserved knot superfamily protein [8–11]. It has a cysteine rich domain, and often presents in soluble or cell-associated forms [12–15]. Its activity could be modified post-translationally via glycosylation and phosphorylation [8, 12–16]. Existing evidences have implied gremlin as an antagonist of bone morphogenetic protein (BMP) [8, 12–16]. It is now known that gremlin participates in several key cellular functions, including cell survival, growth, differentiation, and development [8, 12–16]. Dysregulation of gremlin is observed in cancer and multiple other diseases [8, 12–16].

Recent studies have also tested the potential function of gremlin in regulating vasoproliferation [16]. It is shown that gremlin could act as a direct and potent agonist of vascular endothelial growth factors (VEGF) receptor 2 (VEGFR2) [9, 16], which is the main receptor for VEGF-mediated angiogenic signals [9, 16, 17]. Gremlin binds to VEGFR2, and activates downstream signalings, *i.e.* Akt-mTOR and Ekr-MAPK cascades, which promote cell survival, proliferation and vasoproliferation [16]. In the current study, our results show that gremlin could significantly attenuate UV-induced skin cell damages possibly via activating VEGFR2 signaling.

## RESULTS

### Gremlin protects human skin keratinocytes and fibroblasts from UV radiation

**Figure 1:**
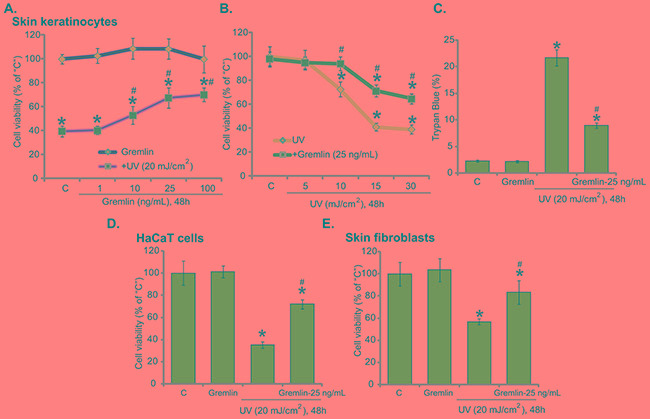
Gremlin protects human skin keratinocytes and fibroblasts from UV radiation Primary cultured human skin keratinocytes **A-C.** HaCaT keratinocytes **D.** or the primary skin fibroblasts **E.** were pretreated with gremlin (1-100 ng/mL) for 30 min, cells were subjected to UV radiation and then cultured in complete medium for additional 48 hours, cell viability was tested by MTT assay (A, B, D and E); Cell death was tested by trypan blue staining assay (C). The values were expressed as the means ± standard deviation (SD) (Same for the following figures). All experiments were repeated at least three times and similar results were obtained (Same for the following figures). “C” stands for medium-treated control group (Same for the following figures). *P < 0.05 *vs.* “C” group. ^#^*P* < 0.05 *vs.* UV only group.

To test the potential effect of gremlin on UV-induced skin cell damages, primary skin keratinocytes were irradiated with UV (20 mJ/cm^2^) with/out gremlin. MTT cell survival assay results showed that UV indeed decreased keratinocyte cell survival, which was largely inhibited with gremlin (10-100 ng/mL) pretreatment (Figure 1A). Gremlin demonstrated a dose-dependent response against UV in keratinocytes (Figure 1A). A low-concentration of gremlin (1 ng/mL) was unable to rescue keratinocytes from UV (Figure 1A). Gremlin at 25 ng/mL showed decent anti-UV activity, and this concentration was chosen for following experiments.

Further studies showed that gremlin (25 ng/mL) protected keratinocytes from UV radiation at other intensities (10, 15 and 30 mJ/cm^2^) (Figure 1B). It also significantly decreased UV-induced keratinocyte cell death, which was tested by trypan blue staining assay (Figure 1C). In HaCaT keratinocytes (Figure 1D) and primary skin fibroblasts (Figure 1E), gremlin (25 ng/mL) pretreatment similarly inhibited UV-induced cell viability reduction. Notably, at tested concentrations (1-100 ng/mL), gremlin alone didn't affect viability or death of above cells (Figure 1A–1E). These results suggest that gremlin protects skin cells from UV radiation.

### Gremlin inhibits UV-provoked apoptosis in skin keratinocytes and fibroblasts

**Figure 2:**
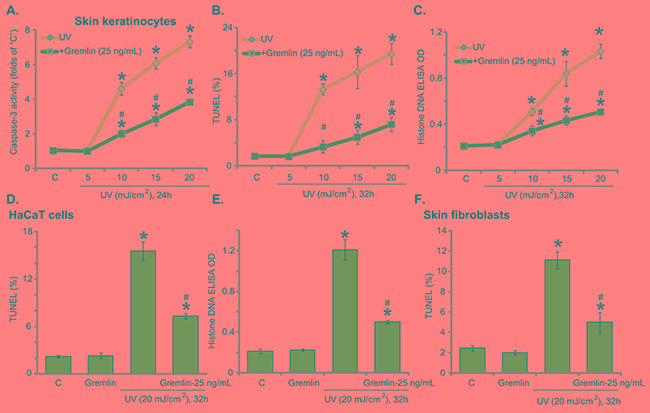
Gremlin inhibits UV-provoked apoptosis in skin keratinocytes and fibroblasts Primary skin keratinocytes **A-C.** HaCaT keratinocytes **D** and **E.** or the primary skin fibroblasts **F.** were pretreated with gremlin (25 ng/mL) for 30 min, cells were subjected to UV radiation (at designated intensity) and then cultured in complete medium for indicated time, cell apoptosis was then tested by listed assays. *P < 0.05 *vs.* “C” group. ^#^*P* < 0.05 *vs.* UV only group.

Next, we tested the potential effect of gremlin on UV-induced cell apoptosis. In line with our previous findings [18, 19], UV-provoked cell apoptosis was tested by the Caspase-3 activity assay (Figure 2A), TUNEL staining assay (Figure 2B) and Histone-DNA ELISA assay (Figure 2C). Results from all these assays demonstrated that UV radiation dose-dependently provoked apoptosis in primary skin keratinocytes (Figure 2A–2C). The caspase-3 activity (Figure 2A), TUNEL percentage (Figure 2B) and Histone DNA ELISA OD (Figure 2C) were all significantly increased following 10-20 mJ/cm^2^ of UV radiation. Remarkably, pre-treatment with gremlin (25 ng/mL) largely attenuated UV-induced apoptosis activation in keratinocytes (Figure 2A–2C), indicating an anti-apoptosis function by gremlin. The similar results were also obtained in HaCaT keratinocytes (Figure 2D and 2E) and primary skin fibroblasts (Figure 2F), where pre-treatment of gremlin (25 ng/mL) significantly inhibited of UV (20 mJ/cm^2^)-provoked cell apoptosis (Figure 2D–2F). Thus, gremlin inhibits UV-provoked apoptosis in skin keratinocytes and fibroblasts.

### VEGFR2 activation mediates gremlin-induced cytoprotection against UV

**Figure 3:**
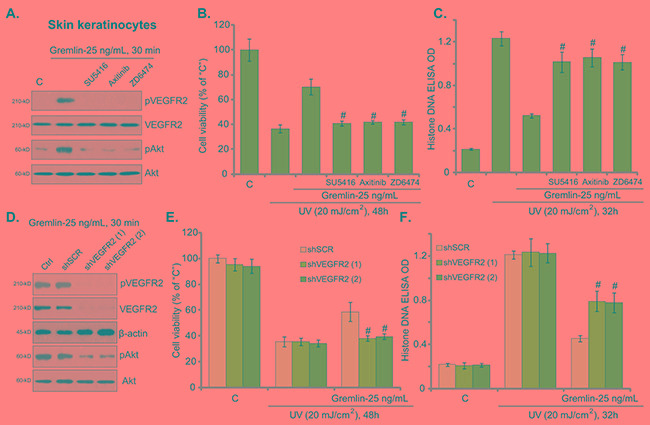
VEGFR2 activation mediates gremlin-induced cytoprotection against UV Primary skin keratinocytes were treated with gremlin (25 ng/mL) or with applied VEGFR2 inhibitors (1 μM of SU5416, 100 nM of Axitinib or 5 μM of ZD6474) for 30 min, expression of listed proteins was tested by Western blot assay **A.** Cells were also subjected to UV radiation, and were cultured in complete medium for applied time, cell viability (**B.** MTT assay) and cell apoptosis (**C.** Histone DNA ELISA assay) were tested. Primary skin keratinocytes expressing the scramble control shRNA (“shSCR”) or different VEGFR2 shRNA (“-1/-2”) were treated with gremlin (25 ng/mL) for 30 min, cells were then subjected to UV radiation and cultured for applied time, indicated protein expression **D.** cell viability **E.** and apoptosis **F.** were tested. “Ctrl” stands for un-transfected cells (D). ^#^*P* < 0.05 *vs.* gremlin group (B and C). ^#^*P* < 0.05 *vs.* gremlin of “shSCR” group (E and F).

Recent studies have proposed that gremlin is a novel VEGFR2 agonist [9, 16]. We tested the possible involvement of VEGFR2 in gremlin-induced activity against UV in skin cells. As shown in Figure 3A, treatment with gremlin in primary skin keratinocytes induced significant VEGFR2 phosphorylation (Tyr1175, the activation site), which was blocked by the known VEGFR2 inhibitors SU5416 [20], Axitinib [21] and ZD6474 [22] (Figure 3A). Further, Akt, the major downstream of VEGFR2 [23], was also activated by gremlin in VEGFR2-dependent manner (Figure 3A). Importantly, co-treatment with the VEGFR2 inhibitors in skin keratinocytes almost abolished gremlin-mediated cytoprotection against UV (Figure 3B and 3C). These results suggest that VEGFR2 activation is required for gremlin's actions against UV in keratinocytes.

To rule out the possible off-target toxicities of the above VEGFR2 inhibitors, shRNA method was applied to knockdown VEGFR2 in keratinocytes. Western blot assay results in Figure 3D showed that VEGFR2-specific shRNAs (“-1” or “-2”, with different sequences) effectively downregulated VEGFR2 in keratinocytes. Gremlin-induced VEGFR2-Akt phosphorylation/activation was also largely inhibited in VEGFR2-silenced cells (Figure 3D). Remarkably, gremlin-mediated cytoprotection against UV was almost nullified in VEGFR2-silenced keratinocytes (Figure 3E and 3F). In another words, gremlin was ineffective against UV when VEGFR2 was silenced (Figure 3E and 3F). Notably, VEGFR2 inhibition or shRNA knockdown alone showed no effect on UV-induced cell death or apoptosis (Figure 3E and 3F, and Data not shown). We repeated these VEGFR2 inhibitor and shRNA experiments in skin fibroblasts, and very similar results were obtained (Data not shown). These pharmacological and genetic evidences suggest that VEGFR2 activation is required for gremlin-mediated cytoprotection against UV in skin cells.

### Activation of Nrf2 signaling, downstream of VEGFR2-Akt, is required for gremlin-induced cytoprotection against UV

**Figure 4:**
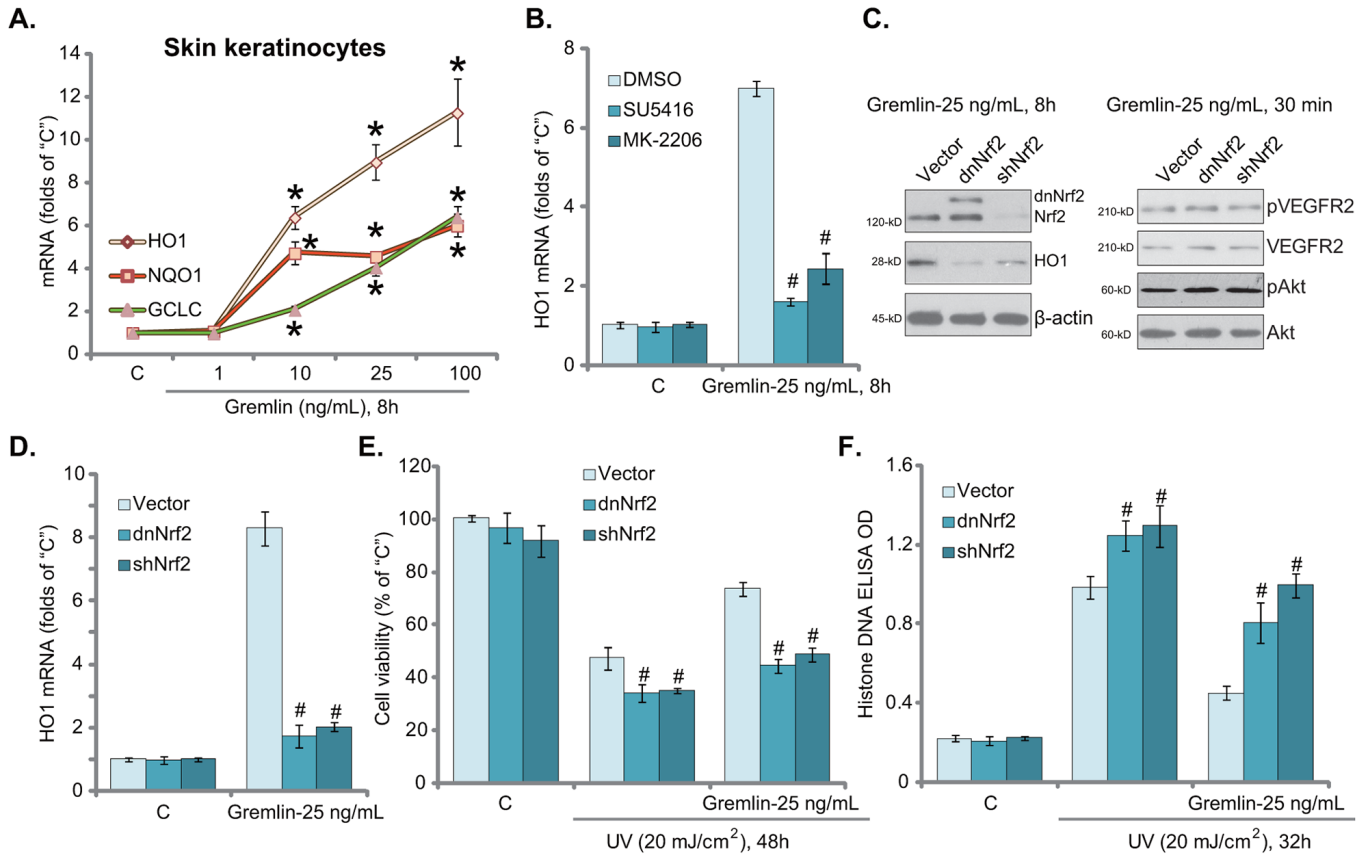
Activation of Nrf2 signaling, downstream of VEGFR2-Akt, is required for gremlin-induced cytoprotection against UV Primary skin keratinocytes were treated with gremlin (1-100 ng/mL) for 8 hours, mRNA expression of listed genes was tested by RT-qPCR assay **A.** Primary skin keratinocytes were pretreated with 1 μM of SU5416 or 10 μM of MK-2206 for 30 min, cells were then treated with gremlin (25 ng/mL) for 8 hours, HO1 mRNA expression was tested **B.** Primary skin keratinocytes expressing the Nrf2 shRNA (“shNrf2”), dominant negative Nrf2 (S40T, “dnNrf2”, Flag-tagged) or empty vector (“Vector”, pSV2 puro-Flag) were treated with gremlin (25 ng/mL) for indicated time, expressions of listed protein and mRNA were tested by Western blot assay **C.** and RT-qPCR assay **D.** respectively. Above cells were also subjected to UV (20 mJ/cm^2^) radiation, or plus gremlin (25 ng/mL, 30 min prior UV), cell viability **E.** and apoptosis **F.** were tested. *P < 0.05 *vs.* “C” group (A). ^#^*P* < 0.05 *vs.* “DMSO (0.1%)” group (B).^#^*P* < 0.05 *vs.* “Vector” group (D-F).

UV-induced ROS production and subsequent oxidative stress is the key cause of subsequent DNA damages and cell apoptosis [24–26]. NF-E2-related factor 2 (Nrf2) is a well-established anti-oxidant signaling [27]. Therefore, we tested the potential activity of gremlin on Nrf2 signaling in skin cells. The real-time quantitative PCR (“RT-qPCR”) assay results in Figure 4A demonstrated that treatment of gremlin in skin keratinocytes dose-dependently increased mRNA expression of Nrf2-regulated anti-oxidant genes, including *heme oxygenase-1 (HO1)*, *NAD(P)H quinone oxidoreductase 1 (NQO1)* and *γ-glutamyl cystine ligase catalytic subunit (GCLC)* [27]. Notably, the VEGFR2 inhibitor SU5416 or the Akt specific inhibitor MK-2206 [28] largely attenuated gremlin-induced HO1 (Figure 4B) and other Nrf2 genes (*GCLC* *and NQO1*, Data not shown) expression. Based on these results, we propose that gremlin possibly activates VEGFR2-Akt downstream Nrf2 signaling in keratinocytes.

To study the involvement of Nrf2 signaling in gremlin-exerted cytoprotection, genetic methods were applied. First, a Nrf2 shRNA (“shNrf2”) was utilized to selectively and stably knockdown Nrf2 in keratinocytes (Figure 4C, left panel). Second, a dominant negative mutation Nrf2 (S40T, “dnNrf2”) [29, 30] was introduced to the keratinocytes (Figure 4C, left panel). As demonstrated, Nrf2 knockdown or mutation largely attenuated gremlin-induced protein and mRNA expression of HO1 (Figure 4C, left panel and Figure 4D) as well as other Nrf2-regulated genes (NQO1 and GCLC1, Data not shown). More importantly, gremlin-mediated anti-UV activity in keratinocytes was largely inhibited with Nrf2 knockdown or mutation (Figure 4E and 4F). Nrf2 knockdown or mutation showed no effect on VEGFR2-Akt activation by gremlin (Figure 4C, right panel). These results suggest that activation of VEGFR2-Akt downstream Nrf2 signaling is important for gremlin-mediated cytoprotection against UV in keratinocytes. Notably, cells with Nrf2 knockdown or mutation were more vulnerable to UV (Figure 4E and 4F), suggesting that basal Nrf2 activation is also important for skin cell survival following UV radiation. The above shRNA and mutation experiments were also repeated in skin fibroblasts, and similar results were obtained (Data not shown).

### Gremlin inhibits UV-induced ROS production and DNA damages in skin keratinocytes and fibroblasts

**Figure 5:**
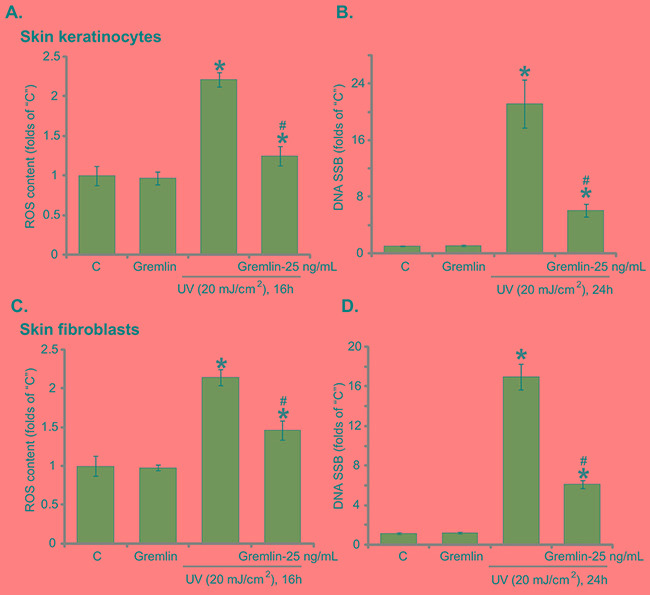
Gremlin inhibits UV-induced ROS production and DNA damages in skin keratinocytes and fibroblasts Primary skin keratinocytes **A** and **B.** or the primary skin fibroblasts **C** and **D.** were pretreated with gremlin (25 ng/mL) for 30 min, cells were subjected to UV (20 mJ/cm^2^) radiation and then cultured in complete medium for indicated time, ROS production (A and C) and DNA single strand breaks (SSBs) (B and D) were tested by listed assays. *P < 0.05 *vs.* “C” group. ^#^*P* < 0.05 *vs.* UV only group.

Excessive UV radiation to skin cells will induce significant DNA damages, mainly single strand breaks (SSBs), which are crucial for the skin cancer transformation [24, 31]. ROS production and oxidative stress are the major causes of DNA damages following UV radiation [24, 31]. Above results showed that gremlin activated Nrf2 signaling, we next tested its role on oxidative stress and DNA damages in UV-irradiated cells. As demonstrated, UV radiation in keratinocytes indeed induced significant ROS production (Figure 5A) and profound DNA SSBs accumulation (Figure 5B), which were largely attenuated with pre-treatment of gremlin (Figure 5A and 5B). The very similar results were also observed in primary skin fibroblasts, where gremlin significantly inhibited oxidative stress (Figure 5C) and DNA SSB formation (Figure 5D) following UV radiation. These results clearly show that gremlin suppresses UV-induced ROS production and DNA damages in skin keratinocytes and fibroblasts.

## DISCUSSION

Excessive UV radiation will induce damages to skin keratinocytes and fibroblasts, causing skin aging or even skin cancer [24, 25, 31]. We here demonstrated that gremlin activated VEGFR2 signaling and significantly attenuated UV-induced death and apoptosis of skin cells (keratinocytes and fibroblasts). Notably, pharmacological or genetic inhibition of VEGFR2 almost abolished gremlin-mediated cytoprotection against UV. Further studies showed that gremlin activated VEGFR2 downstream Nrf2 signaling, which appeared required for subsequent skin cell protection. Nrf2 shRNA knockdown or dominant negative mutation (S40T) largely inhibited gremlin-mediated anti-UV activity in the skin cells.

Studies indicate that UV radiation causes damages to skin cells, which will lead to the formation of so-called “initiated” cells [24, 31, 32]. These cells, often contain DNA injuries, may eventually grow into tumor cells if not eliminated or treated [24, 31, 32]. These initiated cells often divide faster than normal cells and, via the processes of colonel expansion and apoptosis evasion, can possibly transform into cancerous cells [1]. The agents that could inhibit UV-provoked DNA damages or eliminate the initiated cells are being tested to stop or reverse the above cancer transformation process [1, 24, 31, 32]. In the present study, we show that gremlin inhibited UV-induced ROS production possibly via activating the Nrf2 signaling, and significantly attenuated following DNA injuries (SSBs) in skin keratinocytes and fibroblasts. Thus, gremlin may be an ideal agent to prevent or even reverse the formation of initiated skin cells.

Nrf2 is one key anti-oxidant signaling in mammalian cells, and its activity could be modified by multiple signaling proteins, including Erk, p38 and protein kinase C (PKC) [33]. More recent studies have proposed that Akt could also act as an upstream signaling of Nrf2 [29, 30, 34]. Activated Akt was shown to promote Nrf2 activation in certain cell types. For example, Lee *et al.,* showed that sulforaphane-activated Nrf2 signaling requires PI3K-Akt activation [35]. Xu *et al.,* proposed that pyocyanin activates Nrf2 signaling as downstream of PI3K-Akt [36]. Zhang *et al.,* demonstrated that Salvianolic acid A activates Akt-dependent Nrf2-HO1 signaling in retinal cells [34]. More recently, Li *et al.,* showed that 3H-1,2-dithiole-3-thione (D3T) induces Nrf2 phosphorylation at Ser-40 as downstream of Akt, which is required for Nrf2 activation and cell protection [29]. In the current study, we show that gremlin-mediated HO1 (and other Nrf2 genes) expression was largely inhibited by the Akt inhibitor MK-2206 [28], suggest that Akt could be the upstream signaling for Nrf2 activation by gremlin. The detailed underlying mechanisms may warrant further investigations.

In conclusion, our results show that gremlin protects skin cells from UV radiation via activating VEGFR2-Nrf2 signaling. Gremlin could be further studied as a novel anti-UV skin protection agent.

## MATERIALS AND METHODS

### Chemicals and reagents

Gremlin and anti-b-actin monoclonal antibody were obtained from Sigma (Shanghai, China). VEGFR2 inhibitors SU5416, ZD6474 and Axitinib as well as the Akt specific inhibitor MK-2206 were obtained from Selleck (Nanjing, China). All other antibodies utilized in this study were obtained from Cell Signaling Technology (Nanjing, China).

### Cell culture and UV radiation

As previously described [37–39], the primary skin keratinocytes, HaCaT keratinocytes and human skin fibroblasts were maintained in Dulbecco's Modified Eagle medium (DMEM) with 10% FBS and necessary antibiotics, in a CO_2_ incubator at 37^o^C. UV radiation equipments and procedures were described in [37, 40, 41]. All the cell culture regents were obtained from Gibco (Suzhou, China).

### Cell survival and cell death assays

MTT cell viability assay and cell death trypan blue staining assay were described in our previous studies [37–39].

### Quantification of cell apoptosis by ELISA

The Cell Apoptosis ELISA Detection Kit (Roche, Palo Alto, CA) was applied to detect apoptosis of skin cells after indicated treatments. The detailed protocols were previously described [37–39].

### TUNEL assay

TUNEL (Terminal deoxynucleotidyl transferase dUTP nick end labeling) In Situ Cell Death Detection Kit (Roche) was applied to quantify cell apoptosis. Cells were also stained with Hoechst 33342 (Sigma) to visualize the nuclei. Cell apoptosis ratio was calculated by the TUNEL percentage (TUNEL/ Hoechst 33342 ×100%). At least 200 cells in 5 random scope fields per treatment were included to calculate TUNEL ratio.

### Caspase-3 activity assay

The detailed protocol for the caspase-3 activity assay was described early [37]. In brief, following the applied treatment, floating cells and growing cells were combined. The cells were then lysed in 2× caspase lysis buffer [37]. Fifty μg of total proteins per sample were mixed with 2× caspase assay buffer [37] and the caspase-3 substrate: Ac-DEVD-AFC. After incubation, the fluorometric detection of cleaved AFC product was performed on a CytoFluor Multi-Well Plate Reader Series 4000 (PerSeptive Biosystems) using a 400-nm excitation filter and a 530-nm emission filter. The caspase-3 activity of treatment group was always normalized to that of untreated control group.

### Real-time quantitative PCR (“RT-qPCR”) analysis

The Trizol reagents (Invitrogen) [37] were applied to extract RNA [30]. A total of 500 ng RNA per treatment was utilized for the reverse transcription with the 2-step RT-PCR kit (Takara Bio, Japan) [29, 34]. The PCR reaction mixture contained 1× SYBR Master Mix (Applied Biosystem), RNA and 200 nM primers. The ABI Prism 7300 Fast Real-Time quantitative PCR (“RT-qPCR”) assay system (Shanghai, China) was utilized to perform the PCR reactions. The ^ΔΔ^Ct method was applied to quantify mRNA expression. GAPDH was tested as the internal control. All the primers were provided by Dr. Jiang's group at Nanjing Medical University [29, 30].

### Western blot assay

Western blot assay was described in detail in our previous studies [37–39]. After ECL detection of indicated protein, indicated band was quantified and normalized to the indicated loading control via the ImageJ software [42].

### Reactive oxygen species (ROS) detection

In line with our previous studies [37, 38], the fluorescent dye dihydrorhodamine (DHR) was utilized to test cellular ROS content by fluorescence-activated cell sorting (FACS; Beckton Dickinson FACScan, Suzhou, China). The ROS fluorescent intensity of treatment group was always normalized to that of untreated control group.

### Measure of DNA single strand breaks (SSBs)

The detailed protocol for analyzing DNA SSBs was described in our previous study [37]. In brief, following the treatment, cells washed and then lysed with the described lysis buffer [37], which was followed by addition of 1.5 mL SDS-EDTA lysis solution supplemented with 0.5 mg/mL proteinase K (Sigma). DNA was eluted with tetrapropyl-ammoniumhydroxide-EDTA (pH 12.1) containing 0.1% SDS at a rate of 0.125 mL/min. Fractions were collected at 20-min intervals for 2 hours. Filters were treated with 400 μL of HC1 (1 M) for l hour at 60°C, and 0.4 M NaOH was added prior to scintillation counting. SSBs were then quantified to the control level.

### shRNA knockdown and stable cell selection

The two non-overlapping VEGFR2 shRNAs (“VEGFR2 shRNA-1/-2”) were gifts from Dr. Jing Qian's group at Children's Hospital of NanJing Medical University. The lentiviral shRNAs were produced by constructing the GV248 vector (Genepharm, Shanghai, China) with a puromycin resistance gene and targeted VEGFR2 shRNA. The scramble control shRNA (sc-108080) and the Nrf2 shRNA (sc-37030-V) lentiviral particles were purchased from Santa Cruz Biotech (Santa Cruz, CA). Stable knockdown method by targeted shRNA was discussed in detail in our previous studies [37–39]. Briefly, the skin cells were cultured with 60% confluence. Targeted lentiviral shRNA was added to the cultured cells for 36 hours. Cells were then selected by puromycin (5.0 μg/mL) for 96 hours. Afterwards, knockdown of targeted protein (VEGFR2 or Nrf2) was confirmed by Western blot assay.

### Nrf2 mutation

The S40T dominant negative (“dn”) Nrf2 (“dnNrf2”) pSV2 puro-Flag plasmid was a gift from Dr. Jiang at Nanjing Medical University [29, 30]. The dnNrf2 plasmid or the empty vector (pSV2 neo) was transfected to primary skin keratinocytes through Lipofectamine 2000 reagents (Invitrogen). The stable cells with dnNrf2 were selected via puromycin (5.0 μg/mL) for 96 hours [29]. Expression of dnNrf2 (Flag-tagged) was detected by Western blot assay.

### Statistical analysis

In each experiment, a minimum of three wells of each treatment was used. Each experiment was repeated a minimum of three times. All data were normalized to control values of each assay and were presented as mean ± Standard Deviation (SD). Data were analyzed by one-way ANOVA followed by a Scheffe's f-test via the SPSS 16.0 software (SPSS Inc., Chicago, IL). Significance was chosen as *P* < 0.05.
